# Comparison of methods for calculating the health costs of endocrine disrupters: a case study on triclosan

**DOI:** 10.1186/s12940-017-0265-x

**Published:** 2017-06-09

**Authors:** Radka Prichystalova, Jean-Baptiste Fini, Leonardo Trasande, Martine Bellanger, Barbara Demeneix, Laura Maxim

**Affiliations:** 1 Institut des Sciences de la Communication (UMS 3665), CNRS (Centre National de la Recherche Scientifique)/Université Paris Sorbonne/UPMC (Université Pierre et Marie Curie), 20 rue Berbier du Mets, 75013 Paris, France; 2 Sorbonne Universités, CNRS UMR 7221, RDDM, Muséum d’Histoire Naturelle, F-75005 Paris, France; 3 Department of Pediatrics, NYU School of Medicine, 403 E 34th St, New York, NY 10016 USA; 4 School of Public Health, University Sorbonne Paris Cité, EA7348 MOS, Paris, France

**Keywords:** Endocrine disruptor, Triclosan, Health costs, REACH regulation, Socio-economic analysis, Chemical risk, Attributable fraction, Probabilistic risk assessment

## Abstract

**Background:**

Socioeconomic analysis is currently used in the Europe Union as part of the regulatory process in Regulation Registration, Evaluation and Authorisation of Chemicals (REACH), with the aim of assessing and managing risks from dangerous chemicals. The political impact of the socio-economic analysis is potentially high in the authorisation and restriction procedures, however, current socio-economic analysis dossiers submitted under REACH are very heterogeneous in terms of methodology used and quality. Furthermore, the economic literature is not very helpful for regulatory purposes, as most published calculations of health costs associated with chemical exposures use epidemiological studies as input data, but such studies are rarely available for most substances. The quasi-totality of the data used in the REACH dossiers comes from toxicological studies.

**Methods:**

This paper assesses the use of the integrated probabilistic risk assessment, based on toxicological data, for the calculation of health costs associated with endocrine disrupting effects of triclosan. The results are compared with those obtained using the population attributable fraction, based on epidemiological data.

**Results:**

The results based on the integrated probabilistic risk assessment indicated that 4894 men could have reproductive deficits based on the decreased vas deferens weights observed in rats, 0 cases of changed T_3_ levels, and 0 cases of girls with early pubertal development.

The results obtained with the Population Attributable Fraction method showed 7,199,228 cases of obesity per year, 281,923 girls per year with early pubertal development and 88,957 to 303,759 cases per year with increased total T_3_ hormone levels.

The economic costs associated with increased BMI due to TCS exposure could be calculated. Direct health costs were estimated at €5.8 billion per year.

**Conclusions:**

The two methods give very different results for the same effects. The choice of a toxicological-based or an epidemiological-based method in the socio-economic analysis will therefore significantly impact the estimated health costs and consequently the political risk management decision. Additional work should be done for understanding the reasons of these significant differences.

**Electronic supplementary material:**

The online version of this article (doi:10.1186/s12940-017-0265-x) contains supplementary material, which is available to authorized users.

## Background

Socioeconomic analysis (SEA) is currently used in the regulatory process in the European Regulation REACH (Registration, Evaluation, Authorization and restriction of Chemicals, EC/1907/2006), the main objective of which is to manage the risk of dangerous substances. In the framework of REACH, SEA is currently applied within the authorisation and restriction processes. Despite its high potential impact on decision-making, SEA calculations employ very heterogeneous methodological approaches, without clear guidance on how impacts should be calculated and weighted [1, 2].

The most commonly used SEA method is cost-benefit analysis [3], comparing costs and benefits of each particular risk management option. For calculating the benefits (i.e., avoided health costs), the economic literature proposes the method of the population attributable fraction (PAF), using epidemiologic studies as input data [4, 5]. However, the quasi-totality of the data used in the REACH dossiers comes from toxicological studies. Epidemiological data are not available for most of the substances on the market and registered under REACH, even for those which might have effects on health and are intended for regulation. Furthermore, it would be unethical to wait until epidemiological studies show adverse health effects in the exposed population.It is therefore critical to be able to employ a method enabling the use of toxicological data for SEA.

Here we applied the Integrated Probabilistic Risk Assessment (henceforth IPRA) method published by Voet and Slob [6], that integrates probabilistic hazard characterisation based on in vivo studies extrapolated to humans with probabilistic exposure assessment. The method of the PAF provides us a basis for comparison for the results obtained with the IPRA method.

We used triclosan (TCS) as a case study for comparing the two methods for the calculation of the share of the population showing an adverse effect. TCS has widespread use as an antibacterial and antifungal agent in many personal care products used on a daily basis, for example soap, toothpaste, cosmetics, mouthwashes or cleaning supplies [7]. TCS is a suspected endocrine disruptor^1^ [8]. Numerous toxicological studies report adverse effects on thyroid function [9–16], on reproductive organ development notably in male rats, decreased testosterone and sperm production [17–19], lowered pup bodyweight [20], early age of pubertal onset [15, 20] and increased uterine weight [15, 21]. Epidemiological studies reveal most marked endocrine effects of TCS on thyroid function (increased circulating levels of T3) [22] on increased body mass index [23, 24] and advanced pubertal development [25, 26].

TCS has been included in REACH in the Community Rolling Action Programme (CoRAP) listing substances for evaluation (ECHA, CoRAP) - a process aimed at clarifying concerns that the manufacture and/or use of these substances could pose a risk to human health or the environment - due to suspected persistent, bioaccumulative and toxic (PBT) as well as endocrine-disrupting properties [8].

In Europe, TCS was also assessed by the Biocidal Products Committee, that confirmed that TCS is a candidate for substitution because of its toxic and very bioaccumulative properties [27]. No safe use could be demonstrated for the proposed use of TCS. Risk was identified for both surface water and for the non-compartment specific effects relevant to the food chain (secondary poisoning).

## Methods

For identifying the relevant input data for our comparative study of the two methods, we carried an extensive literature review for toxicological, epidemiological and biomonitoring studies. The search method (key words, databases, selection criteria) and the resulting papers identified and finally used in our study are described in detail in the supplementary material (Additional file 1). The studies used in our calculations are in Table 1.

**Table 1:**
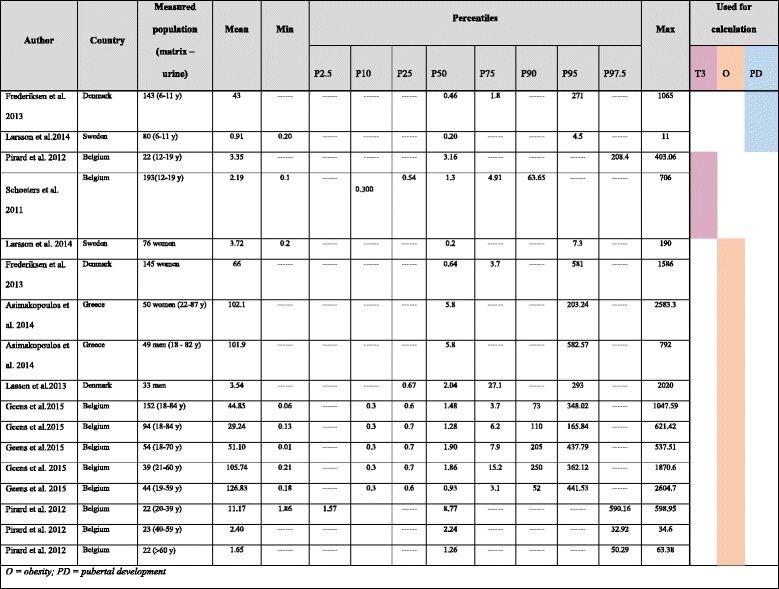
Biomonitoring studies identified for calculation (TCS in ng/mL)

The variable of interest for economists, for the calculation of the total health costs, is the share (percentage), of the total population exposed, that shows a particular effect. Two approaches can be used to estimate this variable: one based on toxicological data (IPRA) and the other on epidemiological data (PAF). The calculation of the total costs is then derived from multiplication by the cost per individual case, which is most often already available in the literature.

### Integrated probabilistic risk assessment (IPRA) model based on toxicological data

For the calculation of the share of the total population exposed showing a given effect, we used the IPRA method described by Voet and Slob [6]. This method is based on toxicological animal data. IPRA integrates a probabilistic distribution of individual critical effect doses (ICED), being the dose associated with a person’s individual Critical Effect Size (CES), with a probabilistic distribution of individual exposures (IEXP). The CES is the percent change in the group mean as compared to the control group mean (e.g., 20% reduction in the acetylcholine-esterase activity) [28]. The Critical Effect Dose (CED) is the dose associated with a particular CES, i.e., the dose where a change (an effect) starts to become adverse [28]. The CED_animal_ was calculated by PROAST, a software tool designed for toxicological data analysis using the Benchmark Dose Modelling (BMD) approach [29]. PROAST includes a set of models (e.g. Weibull, logistic, probit-normal etc.) that can potentially describe a statistical relationship between the dose of a certain chemical and a considered effect (response) of that chemical. Thus, PROAST allows to fit a single dose–response function on the available toxicological data. In case of quantal data, the ED_50_ is calculated instead of CED [28].

The CED_human_ was obtained by application of an interspecies factor (animal to human) and an intraspecies factor (differences between human individuals). All original studies considered in our analyisis used the rat as a model. Therefore, the interspecies extrapolation was done by dividing by a factor of 10, composed of two factors, as recommended by ECHA: a factor of 4 (allometric scaling for rats) and a factor of 2.5 (for toxicokinetic and toxicodynamic differences between animals and humans) [6, 30]. A probabilistic intraspecies factor was calculated in Microsoft excel using the function lognorm.inv. with geometric mean 1, geometric SD 1.98 [6] and 10,000 iterations (Monte Carlo method). Finally, we converted human CED into internal dose, because the exposure data available in the biomonitoring studies was measured in urine (internal), whereas exposure considered in toxicological studies was measured as oral exposure (external). The conversion was done according to formula published by Krishnan et al. [31] for transforming from oral exposure to internal exposure:

1 $$ {\boldsymbol{c}}_{\boldsymbol{v}}=\frac{{\mathbf{D}}^{\ast}{\mathbf{BW}}^{\ast}{\boldsymbol{F}}_{\boldsymbol{UE}}}{\mathbf{V}} $$

where C_v_ is the average urinary concentration on a volume basis of TCS (mg/L); D is the unit dose of TCS (μg/kg/day); BW is the body weight (kg); F_UE_ is the urinary exrection fraction (0.54), and V is the 24-h average urinary volume (litres). This formula makes the assumption that the conversion factor is the same for individuals with or without effect, and does not account for potential differential bias when converting from exposure to urine concentration.

The internal exposure data were collected from publicly available TCS biomonitoring studies (Additional file 1). From this data the estimated mean and estimated SD were calculated. The exposure distribution was assumed as lognormal and randomly calculated for 10,000 iterations, using the @RISK software.

The ratio of these distributions (ICED/IEXP) was calculated in the IPRA method using Monte Carlo analysis and resulted in a distribution of individual margins of exposure (IMoE). The share of the exposed population at risk is the probability that IMoE is lower than 1 (individual IEXP > ICED) [6, 28].

Finally, the human target group was chosen based on approximating the age of tested animals in the human population, according to Sengupta [32].

### Attributable fraction method based on epidemiological data

The attributable fraction is a “*a measure that quantifies the proportion of burden of diseases among exposed people that can be attributed to the exposure”* ([33], p.128). The attributable fraction can be generalized to the total population of exposed and unexposed individuals in order to quantify the importance of the exposure at the population level [34]; the population attributable fraction is “*the fraction of all cases (exposed and unexposed) that would not have occurred if exposure had not occurred*” ([35], p. 508), i.e., the proportion of all cases that can be attributed to a specific exposure [5, 36, 37].

For the calculation of the attributable fraction, different formulas are used in the literature [35, 37, 38]. These formulas vary according to the inputs of exposure – response relationship, which can be relative risk (RR), odds ratio (OR), or the function of exposure response.

**Fig. 1:**
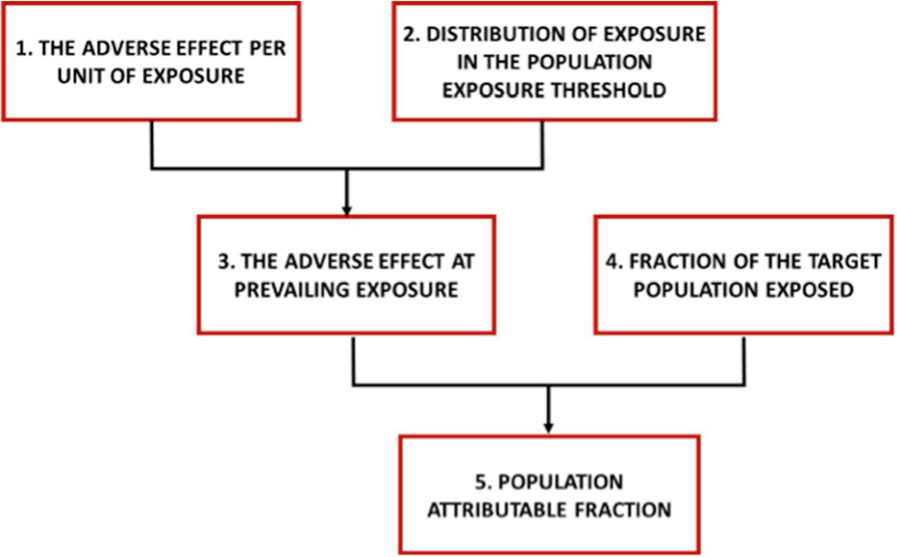
The general steps of PAF calculation

To estimate the health costs related to a specific chemical substance using the population attributable fraction, we followed the approach proposed by Bellanger et al. [4] and Legler et al. [39]. This approach consists of several steps:

- identification of the available epidemiological studies containing an exposure-response relationship between the substance and the health outcome of interest,
- selection of the epidemiological study to use in the calculation
- selection of the target population for the calculation, same as the population studied in the epidemiological study
- identification of exposures based on available biomonitoring studies,
- calculation of the attributable fraction by applying the exposure-response relationship on the exposure percentiles obtained from biomonitoing studies,
- calculation of the case fraction, i.e. the percentiles of the normal distribution of the target population
- calculation of the population attributable fraction from the attributable fraction and the case fraction (see Fig. 1).

#### The adverse effect per unit of exposure

Five epidemiological studies from the United States (US) identified associations between exposure to TCS and health adverse effects of interest [22–26]. The first cross-sectional study assessed the association between urinary TCS and increased T3 hormone in adolescents [22]. The second and third longitudinal^2^ studies assessed the association between urinary TCS and pubertal stage in girls [25, 26]. The fourth study was a longitudinal study examining the association between urinary TCS and elevated body mass index (BMI) in adults [23]. The fifth study was a longitudinal study assessing the inverse association between urinary TCS and BMI in the general population [24]. No European Union (EU)-based epidemiological studies were identified.

#### The distribution of the exposure in the population and the exposure threshold

When an exposure-response relationship was identified for a particular exposure period in a study, this relationship was applied to the EU target population based on the biomonitoring studies selected, presuming that exposure levels in the EU were in the same range as in the US. TCS values were inferred for target populations – children, pregnant women, women, adolescents and adults. We selected the biomonitoring studies where the age of the population measured corresponded to the age of the individuals included in the epidemiological study displaying the health effect. As the exposure measurement units were different for biomonitoring and epidemiological studies, we converted TCS expressed as μg/g creatinine into TCS ng/mL of urine, using the formula for the average volume of creatinine in human urine (10 mmol of creatinine/L of urine) [40]. As the average amount of creatinine is 1.13 mg/mL urine, then 1 g creatinine is ≈ 885 mL of urine:

2 $$ \mathrm{Concentration}\ \mathrm{of}\ \mathrm{substance}\ \left(\mathrm{TCS}\ \mathrm{ng}/\mathrm{mL}\ \mathrm{urine}\right)=\mathrm{Concentration}\ \mathrm{of}\ \mathrm{substance}\ \left(\upmu \mathrm{g}/\mathrm{g}\ \mathrm{creatinine}\right)/885\ \mathrm{mL}\ \mathrm{urine} $$

The biomonitoring studies reported the levels of TCS in different manners. Some of the studies only provide the mean, others the 5th, 25th, 50th, 75th, 90th, 97.5th percentiles and the maximum. Others provide the median TCS level and the interquartile ranges (see Table 1). To obtain consistent data for our calculations (mean and standard deviation, SD), when certain parameters were not available, they were estimated from the data provided as described below.

If the mean was not given, the median was used. SD was calculated according to the percentiles and z-score of highest percentile, (see formula 3; (Z-score, Chart table)). Z score is the value on x-axis and y-axis under the specified percentile possible to find in the z-score chart table.^3^

3 $$ \boldsymbol{SD}=\frac{\mathbf{concentration}\ \mathbf{of}\ \mathbf{TCS}\ \mathbf{in}\ \mathbf{the}\ \mathbf{highest}\ \mathbf{percentile}-\mathbf{the}\ \mathbf{concentration}\ \mathbf{of}\ \mathbf{TCS}\ \mathbf{in}\ \mathbf{the}\ \mathbf{P}50}{\mathbf{z}-\mathbf{score}\ \mathbf{of}\ \mathbf{the}\ \mathbf{highest}\ \mathbf{percentile}} $$

The estimated mean and estimated standard deviation were calculated in the following way:

1. We calculated the natural logarithm for each available biomonitoring study relevant for the effect considered, separately: ln(mean), ln(SD^2).
2. Then, logarithm of mean and SD were multiplied by the size of the population in the study (N): N*ln(mean), and N*ln(SD^2).
3. The average ln(mean) was calculated as the sum of N*ln(mean) for all the relevant biomonitoring studies, divided by the sum of the sizes of the populations measured in the studies (formula 4).
4. The exponential function was used to obtain the estimated mean (formula 5).

4 $$ \mathbf{ln}\left(\mathbf{mean}\right)\to {\mathbf{N}}^{\ast}\mathbf{ln}\left(\mathbf{mean}\right)\to \mathrm{Average}\  \ln \left(\mathrm{mean}\right)=\frac{\sum \left( \ln {\left(\mathrm{mean}\right)}^{\ast}\mathrm{N}\right)}{\Sigma \mathrm{N}} $$

5 $$ \boldsymbol{Estimated}\ \boldsymbol{mean}=\boldsymbol{exp}\left(\boldsymbol{averageln}\left(\boldsymbol{mean}\right)\right) $$

5. The estimated SD was calculated as in formulas 6 and 7.

6 $$ \mathbf{ln}\left({\mathbf{SD}}^{\wedge \mathbf{2}}\right)\to {\mathbf{N}}^{\ast}\mathbf{ln}\left({\mathbf{SD}}^{\wedge \mathbf{2}}\right)\to \mathrm{Average}\  \ln \left({\mathrm{SD}}^{\wedge 2}\right)=\frac{\sum \left( \ln {\left({\mathrm{SD}}^{\wedge 2}\right)}^{\ast}\mathrm{N}\right)}{\Sigma \mathrm{N}}\to \exp \left(\mathrm{average}\  \ln \left({\mathrm{SD}}^{\wedge 2}\right)\right) $$

7 $$ \boldsymbol{Estimated}\ \boldsymbol{SD}=\sqrt{\boldsymbol{exp}\left(\boldsymbol{average}\ \boldsymbol{ln}\left(\boldsymbol{SD}\wedge 2\right)\right)\ } $$

6. In the final step, we calculated the different percentiles of the exposure distribution within the population. For this, we used the estimated mean, estimated SD and z-score for each percentile according to the formula 8:

8 $$ {\boldsymbol{P}}^{\boldsymbol{i}}=\mathbf{Estimated}\ \mathbf{mean}+{{\boldsymbol{z}-\boldsymbol{score}}^{\boldsymbol{i}}}^{\ast}\boldsymbol{Standard}\ \boldsymbol{Deviation} $$

A threshold value, i.e., a level of TCS above which the adverse effect, meaning the increase in T3, was significant, was not available for TCS and T3. Such a value could be derived from toxicological studies, using the BMD approach. However, we considered that this would introduce bias into the methodological comparison between toxicological and epidemiological studies. Therefore, for the calculations of the attributable fraction we only included values from the selected epidemiological studies and made the assumption that the first reported percentile was the threshold (P25 in our case, see Additional file 2).

#### Adverse effect at prevailing exposure (attributable fraction)

Calculations of the attributable fraction can be done using two formulas, i.e. with (case 1) or without a threshold value (case 2).

##### Case 1: With exposure threshold

The threshold value and the exposure-response relationship were applied to the exposure to the substance in each percentile, using the function “IF” from Excel (formula 9). If the concentration of the substance (TSC) is higher than the exposure threshold to TCS, then the variation in the response (e.g., percentage increase in T3) was multiplied by the ratio between the substance concentration and the variation in the dose of the substance provided in the study (e.g.: an increase of an inter-quartile range) (Koeppe et al., [22]). We obtained the variation in the response corresponding to the variation in the dose considered.

9 $$ \mathbf{IF}\left(\mathbf{concentration}\ \mathbf{of}\ \mathbf{substance}>\mathbf{threshold}\ \mathbf{value};\mathbf{variation}\ \mathbf{of}\ \mathbf{the}\ {\mathbf{response}}^{\ast}\left(\mathbf{concentration}\ \mathbf{of}\ \mathbf{substance}/\mathbf{variation}\ \mathbf{in}\ \mathbf{the}\ \mathbf{dose}\ \mathbf{of}\ \mathbf{the}\ \mathbf{substance}\right);\mathbf{0}\right) $$

Afterwards, the attributable fraction for the response considered (i.e., the proportion, among the exposed individuals,that showed a response because they were exposed to the substance) was calculated with the norm.dist function according to formula 10:

10 $$ \mathbf{Attributable}\ \mathbf{fraction}=\mathbf{1}\mathbf{\hbox{--}}\mathbf{NORMDIST}\left(\mathbf{upper}\ \mathbf{normal}\ \mathbf{level}\ \mathbf{of}\ \mathbf{the}\ \mathbf{response},\mathbf{Mean}\ \mathbf{level}\ \mathbf{of}\ \mathbf{response}\ \mathbf{in}\ \mathbf{the}\ {\mathbf{population}}^{\ast}\%\mathbf{in}\mathbf{crease}\ \mathbf{in}\ \mathbf{the}\ \mathbf{response}+\mathbf{Mean}\ \mathbf{level}\ \mathbf{of}\ \mathbf{response}\ \mathbf{in}\ \mathbf{the}\ \mathbf{population},\mathbf{SD},\mathbf{true}\right) $$

##### Case 2: Without exposure threshold

When threshold value was not available, the calculations exploited the IF function. All the other steps are the same, except when the association is expressed as the hazard ratio (HR).^4^ In this latter case, the calculation is carried out as the (RR-1)/RR [37]. This formula is applied on each percentile, and results in the attributable fraction.

#### The fraction of the target population exposed

To obtain the fraction of the target population exposed, we searched - within the available databases in Europe - the size of the population (exposed and unexposed) corresponding to the age considered in the epidemiological study. Then we divided this target population into percentiles.

#### The population attributable fraction

The final population attributable fraction, is calculated according to the formula 11.

11 $$ \boldsymbol{PAF}={{\boldsymbol{AF}}_0}^{\ast}{\boldsymbol{CF}}_0+{{\boldsymbol{AF}}_1}^{\ast}{\boldsymbol{CF}}_1+{{\boldsymbol{AF}}_2}^{\ast}{\boldsymbol{CF}}_2\dots +{{\boldsymbol{AF}}_{\boldsymbol{n}}}^{\ast}{\boldsymbol{CF}}_{\boldsymbol{n}} $$

where CF_i_ (*i* = 0 to n) is a percentile range of the target population (calculated from available databases) and AF_i_ (*i* = 0 to n) is the attributable fraction corresponding to that percentile range.

## Results

With the IPRA method, we calculated the share of population showing an adverse effect for three different health endpoints related to TCS: decreased vas deferens weight as an indication of adverse effects on testicular funtion, decreased T3, and early onset of vaginal opening as an indication of precocious puberty. The results of the calculations are given below.

**Decreased vas deferens weight** in rats was identified as an adverse effect on testicular function reported by Kumar et al. [18]. The CES used 5% as default value for continuous data [6].

**Fig. 2:**
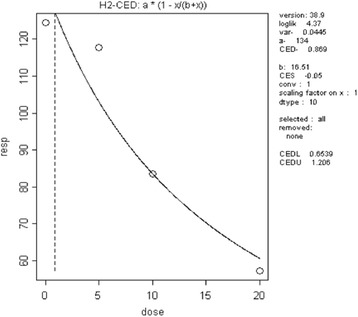
Dose response relationship for vas deferens weight (vertical axis, mg) and TCS (horizontal axis, mg/kg bw). The parameter of interest (right side) is CED. The formula of the dose–response relationship is above the graph, H2 indicates the Hill model

The CED_animal_ was calculated using the PROAST software (Additional file 3). The final curve and its parameters are presented in Fig. 2.

The CED_animal_ obtained for decreased vas deferens weight was 0.869 mg/kg bw day. After the application of interspecies factor, the CED_human_ was 0.0869 mg/kg bw day. Exposure data are published as TCS concentrations in human urine. As CED_human_ was based on toxicological data measured as external exposure (oral, in mg/kg/day given to the experimental animals), we had to convert this into mgTCS/urine (L). The conversion was done according to the following formula:

$$ {\mathrm{C}\mathrm{ED}}_{\mathrm{human}}\ \left(\mathrm{internal}\right)={\mathrm{C}}_{\mathrm{V}}=0.086{9}^{\ast }3{2}^{\ast }0,54/0.66 $$

The CED converted into internal was 2.28 mg/L. This value was then divided by the probabilistic intraspecies factors, which resulted in the probabilistic CED_human_ used in calculation of the share of the target population concerned by the effect. Random ICED were divided by random IEXP, which resulted in the share of the population for which IEXP was higher than ICED.

Using exposure data from two biomonitoring studies [41, 42], the estimated mean and estimated SD were 0.01 mg/L and 0.05 mg/L, respectively. The distribution was truncated to the range between 1st percentile and 99th percentile.^5^ The target population was chosen with relation to age of the rats used in the toxicological study.

The calculated share of the target population (5–10 years old boys) showing the considered adverse effect was equal to 0.03% (Additional file 2, sheet IPRA VD).

The total number of boys (5–10 years old) was taken from EUROSTAT, i.e., 16,314,864 in 2014. The share of population obtained from calculation was applied on this total number.

The final result was 4894 cases^5^, which would reflect number of boys in Europe showing a modification of testicular function due to exposure to TCS.

**Fig. 3:**
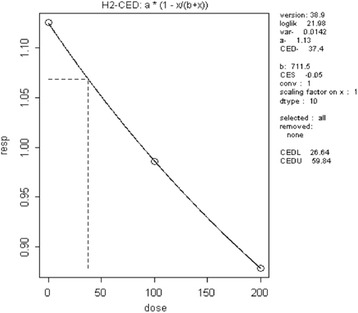
The dose response relationship for decreasing circulating T3 hormone (vertical axis) and TCS (horizontal axis)

**Decreased T3 hormone** was reported by Zorilla et al. [16]. Based on the same methodological steps as for decreased vas deferens weight, and a CES of 5% used as the default value for continuous data [6], the CED_animal_ was calculated using the PROAST software (Additional file 4). The final dose–response relationship and its parameters are presented in Fig. 3.

The calculated CED_animal_ for T3 was 37.4 mg/kg bw day. After the application of the interspecies factor of 10, the CED_human_ was 3.74 mg/kg bw day.

The exposure data are published as concentration of TCS in human urine. Therefore CED_human_ (mg/kg) bw was converted into mg TCS/urine (L). The conversion was done as follows:

$$ {\mathrm{C}\mathrm{ED}}_{\mathrm{human}}\ \left(\mathrm{internal}\right)={\mathrm{C}}_{\mathrm{V}}=3.7{4}^{\ast }5{7}^{\ast }0.54/1.65 $$

This internal CED used in the calculation was 69.77 mg/L. Using exposure data from one biomonitoring study [43], the mean and SD were 0.00219 mg/L and 0.08203 mg/L, respectively. The distribution was truncated within the entered minimum and maximum range taken from the original biomonitoring study.^6^ The target population was chosen in relation to the age of the rats used in the toxicological study, i.e., 12–19 years old girls and boys. The calculated share of the target population concerned by the adverse effect was equal to 0 (Additional file 2, sheet IPRA T3). The total number of 12–19 years old adolescents was taken from EUROSTAT, i.e., 43,003,188 in 2014. The share of population obtained from calculation was applied on this total number, which resulted in 0 individuals concerned by this effect.

**Fig. 4:**
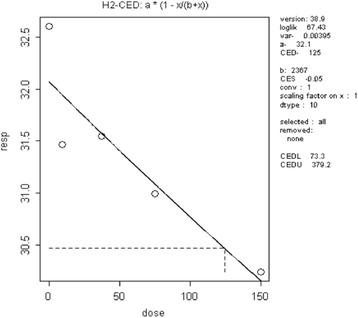
The dose response relationship for early onset of vaginal opening (vertical axis, age) and TCS (horizontal axis, mg/kg)

For **early onset of vaginal opening**, the toxicological study used [15] shows an effect of TCS on pubertal development, i.e. on early onset of vaginal opening. Based on the same methodological steps as above and a chosen CES of 5% used as the default value for continuous data, the CED_animal_ was calculated using the PROAST software (Additional file 5 and Fig. 4).

The modelled CED_animal_ was 125 mg/kg bw day, leading to a CED_human_ of 12.5 mg/kg bw day after application of the interspecies factor.

The exposure data are published as concentrations of TCS in human urine. Therefore CED_human_ in unit mg/kg bw had to be converted into mg TCS/urine (L). The conversion was calculated as follows:

$$ \mathrm{CEDhuman}\ \left(\mathrm{internal}\right)={\mathrm{C}}_{\mathrm{V}}=12.{5}^{\ast }3{2}^{\ast }0.54/0.66 $$

This internal (converted) CED used in the calculation was 327.27 mg/L.

The exposure data were retrieved from two biomonitoring studies [41, 42]. The estimated mean and estimated SD were 0.01 mg/L and 0.03 mg/L, respectively. The distribution was truncated to the range between 1st percentile and 99th percentile. The target population was chosen with relation to age of the rats used in the toxicolgical study and was 6–8 years old girls. The total number of girls was taken from EUROSTAT, i.e. 7,761,173 in 2014. The calculated share of population concerned by the adverse effect was null (0%) (Additional file 2, sheet IPRA PD). This share was applied on the total number and resulted in 0 individuals concerned by this effect.

**For the method of Attributable Fraction for increased T3 in adolescents**, we used a study that observed a 3.8% increase of total T3 per interquartile range (IQR) of increased TCS concentration in the urine, in adolescents (males and females together) [22]. Two biomonitoring studies measured exposure of adolescents within EU. We used one study for our calculation [43]. As the second study measured a very small sample, we conducted a sensitivity analysis to see how this small sample can influence the final results [44].

We used the original distribution of exposures reported in the study [43]. The exposure level within each percentile is shown in Table 2 (Additional file 2, sheet AF T3; cells E10:J10).

**Table 2:** The attributable fraction of increased T3

Percentiles		P0–9	P10–24	P25–49	P50–74	P75–89	P90+
Concentration of TCS (ng/mL)		0	0.3	0.54	1.30	4.91	63.65
Share of population that shows the effect (increased T3)	Boys	0	0	0	0	0.000217	0,015093
	Girls	0	0	0	0	0.000102	0.026084
Total number of cases	88,957						
Sensitivity analysis							
Total number of cases	303,759						

We assumed that the threshold value is the lowest percentile of TCS concentration (P25) provided in the longitudinal study [22]. We used this threshold for male and female exposure separately because they were distinguished in the original study. The measurement unit from the original study (μg/g creatinine) was converted into ng/mL of urine. The final threshold TCS values were 3.84 ng/mL for males and 4.62 ng/mL for females.

The upper normal levels of circulating T3 were retreived from the literature, being for girls and boys 192 ng/dL, and 195.3 ng/dL respectively [45]. We used these values to calculate the attributable fraction, using norm.dist function (see Attributable fraction method based on epidemiological data). The results are summarised in Table 2.

Male and female adolescents were our target group. The target population was considered to be normally distributed and divided into percentiles.

Next, all the percentiles were summed (according to formula 11 The population attributable fraction) and the final number of all increment cases (males and females together) was estimated as 88,957 to 303,759 cases per year, see Table 2.

We carried out a sensitivity analysis to see how the final results could be influenced by the size of population considered in the available biomonitoring studies. For the sensitivity analysis we included a second biomonitoring study [44], initially excluded because of its small cohort size (22 participants). The calculated number of individuals showing increased T3 hormone due to TCS exposure reached 303,759 cases per year.

**Calculation of the Attributable Fraction for increased BMI in adults** used an association between urinary TCS and body mass index (BMI) found in the literature. Such an association was published in three epidemiological studies. One study found an inverse association between TCS and BMI [24], and two other studies found a positive association between TCS and an increase in the BMI (Geens et al., [46]; Lankester et al., [23]). We excluded one study [46] because it measured this association only in obese people, and another one because it did not include a non-exposed population [24]. We selected the longitudinal study [23], in which the authors measured the exposure-response relationship within the whole adult population. The exposure response relationship was non-linear and TCS was associated with significant increases of 1.53 and 1.04 BMI points in the second and third quartiles.

The exposure data for adults were collected from six biomonitoring studies [41, 42, 44, 46–48] (Table 1). The measurement units were converted into common unit, ng/mL. Estimated mean and estimated SD were calculated, and their final values were 31.21 ng/mL and 121.18 ng/mL respectively.

The exposure levels within each percentile is shown in Table 3 (Additional file 2, sheet AF BMI; cells E14:J14).

**Table 3:** The attributable fraction for obesity

Percentiles		P0–9	P10–24	P25–49	P50–74	P75–89	P90+
Concentration of TCS (ng/mL)		0	0	0	31.21	112.40	186.32
Increase of BMI points		0	0	0	1.04	0.26	0.26
Prevalence obesity %	Women	0	0	0	5.72	1.37	1.37
	Men	0	0	0	6.25	1.52	1.52
Direct health costs per case	€ 811						
Total number of cases	7,199,228						
Total direct health costs	€ 5,838,573,648						
Sensitivity analysis							
Total number of cases	7,199,228						
Total direct health costs	€ 5,838,573,648						

Based on mean BMI values in each of the EU countries identified from previous publications [39, 49], we calculated the mean BMI for the European Union separately for men and women. The estimated mean BMI for men and women in Europe was 26.8 and 25.79 respectively, which we used for the calculation of the obesity prevalence (i.e. the number of obese people). According to WHO, obesity is defined as a BMI greater than or equal to 30. This obesity threshold was used in the calculation of obesity prevalence, using the norm.dist function in excel (see formula 10).

We summed the appropriate increments of BMI point for each exposure percentile. Finally, we subtracted the obesity prevalence from the increment of BMI points for each percentile of exposure, which resulted in the attributable fraction for each percentile of exposure. The results are summarised in Table 3 (Additional file 2; sheet AF BMI; cells G23,24:L23,24).

Our two target groups were adult men and women (20 to 85 years old). The size of each target population was divided into percentiles and multiplied by the corresponding attributable fraction.

After this, the case fractions for all percentiles were totalled and the final number of cases of obesity was estimated as 7,199,228 individuals per year (1,85% of the target population).

Direct annual health costs were published by Lengerke and Krauth [50] and equaled €811 per case per year. The final health costs due to exposure to TCS were calculated as number of cases*the cost per case, reaching €5.8 billion per year.

Initially, we excluded pregnant women from the biomonitoring studies measuring the adult population, because during pregnancy women exhibit altered metabolism that physiologically impacts their BMI. We conducted a sensitivity analysis for understanding how the results were influenced by this choice. When exposure data for pregnant women [51–53] was included in the calculations, the number of individuals within each percentile showing obesity due to their exposure to TCS did not change (7,199,228 individuals).

**Calculation of the Attributable Fraction for early pubertal development** used an epidemiological study [26], which published a HR for 5th quintiles of TCS urinary concentrations equal to 1.17.

The exposure data for girls was taken from two biomonitoring studies that measured TCS in 6–11 years old children [41, 42] (Table 1). The mean and estimated SD (the study published only the mean therefore SD needed to be estimated) were 11.08 μg/g creatinine and 43.93 μg/g creatinine respectively. The exposure level within each percentile is shown in Table 4 (Additional file 2; sheet AF PD; cells C15:H15).

**Table 4:** The attributable fraction for early pubertal development

Percentiles	P0–9	P10–24	P25–49	P50–74	P75–89	P90+
Concentration of TCS (μg/g creatinine)	0	0	0	11.08	40.51	67,30
HR at prevailing exposure	0	0	0	1	1.17	1.17
Total number of cases	281,923					
Sensitivity analysis						
Total number of cases (based on [41] alone)	112,769					
Total number of cases (based on [42] alone)	0					

As the increase in HR has been published for a range of TCS concentration, we assigned to each percentile range of TCS concentration, the corresponding HR. To obtain the attributable fraction, we applied the formula (HR-1)/HR [54].

This study looked at the association between TCS and early pubertal development in 6–8 years old girls, which was our target group. We divided this population into percentiles and multiplied them by the corresponding attributable fraction, for obtaining the case fractions for each percentile. The case fractions for all the percentiles were totalled and the final number of cases was estimated as 281,923 individuals (Table 4).

We conducted a sensitivity analysis to determine whether the results were influenced by using both biomonitoring studies for the calculation. When only the exposure data from Frederiksen et al. [41] was used in the calculations, the number of total cases was 112,769 (Additional file 2, sheet AF PD SA Frederiksen). When the exposure data from Larsson et al. [42] was used in the calculations, the number of total cases was 0 (Additional file 2, sheet AF PD SA Larsson).

## Discussion and conclusions

Socioeconomic analysis is currently used as part of the regulatory process in the European Regulation REACH, aiming at assessing and managing risks from dangerous chemicals. Whereas the political impact of SEA is potentially high in the authorisation and restriction procedures, current regulatory SEAs are very heterogeneous in their methodological choices and quality [55]. Furthermore, the economic literature is not very helpful as most published calculations of health costs associated with chemical exposure use epidemiological studies as input data, whereas the quasi-totality of the data used in the REACH dossiers comes from toxicological studies.

The comparison of the results obtained from both methods (Table 5) was done for two endpoints addressed in both toxicological and epidemiological studies, namely the variations in the active form of thyroid hormone, trio-iodothyronine or T_3,_ and early pubertal development. Our main finding is that the two methods gave markedly different results. Therefore, clearly, the choice of the method to be used in regulatory SEA, i.e., toxicological-based or epidemiological-based, is crucial as, in turn, it will have significant impacts on the estimated health costs and subsequent decision-making.

**Table 5:** Results obtained from the two methods

Adverse effect	Number of cases	
	IPRA	PAF
Decrease in vas deferens weight/adverse effect on testicular function	4894	--------
Increased T3 levels	0	88,957–303,759
Early pubertal development	0	281,923
Obesity	--------	7,199,228

Taken together, our results emphasise that more research is required before the toxicological-based modelling methods in SEA can be used. Most importantly, a second step including uncertainty assessment has to be considered in further work, as recommended by Voet and Slob [6]. Additional calculations are needed to identify which inputs impact most significantly on results. Further, potential methodological drawbacks should be clarified before using the IPRA method for regulatory ends. Indeed, BMD modelling uses numerous assumptions [56] which can strongly influence the use of BMD-based methods, including the IPRA method used here, for calculating the share of the population susceptible that shows a negative effect related to exposure to TCS or another chemical. Such analysis is critical given the current increasing tendency to use such models for regulating chemical risks in Europe, where they can be used as “black boxes” that give needed figures but are not understood in their inner structures and assumptions [56].

Most probably, the differences between the two methods come from the numerous assumptions behind the probabilistic modeling, including extrapolation from animal to human when using toxicological data and from remaining uncertainties in epidemiologic studies.

Indeed, probabilistic modeling including BMD can be significantly influenced by subjective expert judgment and assumptions behind the tool itself and its recommended use [56] e.g., the choice of the 5% as typical level of significance used to choose the best-fitted curve, the criteria used for choosing the best-fitted model (acceptability, similarity with the log-likelihood with the full model), the choice of the BMR (Benchmark Response) of 5% (whereas levels of 1% to 10% can be chosen and have been reported in the literature). Furthermore, the BMD results depend of the sample size of the original studies, i.e. with increasing sample sizes, BMD tends to increase. Even if we have explicitly tried to select the best available toxicological studies, the number of doses in the toxicological studies available for our case studies was relatively small as referred to their statistical use in the BMD modeling, regardless of biological limits and type of expected response curves. This feature is indeed a very common characteristic of toxicological studies and it does present a difficulty for the regulatory use of the BMD method in general.

There is still no standardized method for applying BMDs, no uniform definition for it and no standardized requirements for the BMD software available. Different definitions of BMD may include specified increase in the probability of an adverse response, specified increase in the probability of an adverse response relative to the probability of a non-adverse response in unexposed subjects, specified change in the mean response, specified change in the mean response relative to the standard deviation, or specified percent change in mean response. In Proast, the BMD used is the dose level, derived from the estimated dose–response curve, associated with a specific change in the response (the BMR); the confidence interval for the BMD accounts for the statistical uncertainty in the estimate of the BMD [57].

Also, the IPRA method is based on one rat study per endpoint, which might be considered as much weaker evidence compared to a long-term, well-designed epidemiological study. Furthermore, IPRA makes use of uncertainty factors, which are not used in PAF.

Extrapolation from animal to human is a widely and continuely disputed issue in the literature and the regulatory arena. For pragmatic reasons related to the need of using non-human data in regulatory chemical assessment, current practice uses assessment factors. As our purpose was not to argue in favor of one or another assessment factor, and as our methodological comparison is relevant for the regulatory arena, we used the values recommended in regulatory practice in Europe. However, we recognize that these (and other) assessment factors values for extrapolation from animal to human are arbitrary [58].

Furthermore, the results of both methods, and the subsequent difference between them, can be influenced by the availability of the toxicological and epidemiological studies. Even if we aimed at selecting the highest quality studies among those available, there is no “perfect study”. Inevitably, the quality of the input data influences the magnitude of the modeled results. For example, the result of epidemiologic studies is a direct input for the AF method but depends on many choices in the research protocol, e.g., related to the appropriateness of the control group(s), the groups number and size, the sampling method, the control of confounders, choice of the parameters measured for detecting effects and their representativeness of the effect measured, selection of the observation time compared to the real potential time range of the effects, the choice of the analytical method for measuring exposure and the statistical test(s) used to analyze the results, the choice of the target group, the timing of sampling, etc.

Furthermore, other endocrine disrupting chemicals could act as confounders. Whereas co-exposure to other chemicals were accounted for in all the five epidemiological papers selected, the list of compounds measured differed, going from only one (BPA, in Li et al. [24]) to 60 (in Lankester et al., [23]). For applying the two methods, we had to make several assumptions to be able to use the existing published data. Thus, exposure mean and SD had to be estimated when they were not reported in the biomonitoring studies and exposure data was assumed to be lognormal for use in the IPRA method. When a threshold was not available, we also had to assume that it was equal to the first reported percentile or the lowest measured concentration of substance. Similarly, we had to convert available external exposure data into internal. Even if the formula used was developed by Krishnan et al. specifically for triclosan, it is still an approximation that can differ more or less from measured data. Also, as mentioned above, internal exposure data were taken from published TCS biomonitoring (Additional file 1). However, modelling could also be used for calculating exposure for substances lacking biomonitoring data.

For the only endpoint for which costs could be calculated, namely obesity, the results are in line with recent work [4, 5, 39] showing the paramount role of endocrine disruption for the health of Europeans and for the EU’s economy. We calculated costs of obesity due to TCS exposure at €5.8 billion per year,. This figure adds to the costs previously calculated for three other chemicals [39]: €24.6 million (social costs) associated with dichlorodiphenyldichloroethylene (DDE) exposure linked to excess weight at age 10, as well of €15.6 billion (direct and indirect costs) associated with phthalate- associated obesity in adult women and lifetime annual social costs of €1.54 billion for obesity associated with prenatal BPA exposure.

Whereas we could not apply the PAF method on vas defererens weight due to a lack of epidemiological data for a similar endpoint in human TCS exposure, previous calculations show that economic costs of endocrine disruption-related impacts on human male reproductive health are high. For an etiological fraction of 20% (i.e., the fraction of incidences assumed to be caused by exposure to endocrine disruptors), the estimated cost of illness related to negative effects on male reproduction due to the current EDC exposure in Nordic countries reached €36 million per year of exposure (in discounted costs, excluding intangible costs of infertility). In the EU-28, the discounted socio-economic costs due to yearly exposure to endocrine disruptors was calculated at €592 million [59]. In a separate study addressing only two chemicals, phthalates and the flame retardant PBDE, the authors estimated male infertility and related health costs at €15 billion per annum for the EU [60].

For other endpoints considered (e.g., modulations in circulating thyroid hormone T_3_), it was not possible to specify specific diseases that would have epidemiological data in health records. Nevertheless, even if they are diffuse and with multiple interlinked consequences, it is clear that such effects do exist. For example, changes in thyroid hormone availability will impact most physiological sytems, including brain function (memory, attention span, mood etc.), reproductive health and metabolic status (with effects on body weight). Effects of higher circulating T_3_ can include precocious puberty (the onset of menarche before 9 years old and the appearance of secondary sex characteristics before 8 years old), with significant health, social and psychological costs in families with children affected) [61]. Effects of small variations, both increases and decreases, in maternal thyroid hormone during early pregnancy can significantly affect children’s IQ and brain structure [62].

The difficulty to associate most endpoints addressed in the toxicological and epidemiological literature on endocrine disruption with public health endpoints raises the question of the choice of appropriate endpoints in research. We suggest that researchers should emphasise the links between the endpoints investigated with public health issues. Such actions which would significantly increase the relevance of their findings for decision-making and amplify impact, providing a “Matthew effect” as seen for other forms of environmental research [63].

To date, health effects which have been suggested as linked to TCS exposure in the population include breast cancer in adult women [64] a feature that could be related to early pubertal development [65]. TCS has also been related to cardiovascular diseases [66, 67]. Both effects could implicate changes in thyroid hormone homeostasis and physiology. Many aspects of human fertility and infertility are related to or regulated by thyroid hormone [68, 69] and timing of puberty is modified in cases of thyroid disfunction [70]. Furthermore, a number of epidemiological studies link both hyper and hypothyroidism to risk of cardiovascular disease either as function of increased BMI or independently of changed BMI [71]. Furthermore, thyroid hormone homeostasis is major factor affecting longevity [72].

The data from the Lankester et al. [23] and Li et al. [24] studies on TCS and BMI fit with the results from [22] Koeppe et al., on TCS exposure and thyroid hormone changes. Thyroid hormone avaibility determines metabolic rate and both hypothyroidism and hyperthyroidism are characterized by marked changes in BMI [73]. In turn, BMI is positively linked to risk of cardiovascular disease. More research could hence be focused on two areas. First, there is a need to establish data on circulating thyroid hormone levels as a function of TCS exposure in the adult population as the data currently available are limited to adolescents. Second, we need more perspective on TCS levels its potential association with cardiovascular problems.

Our results support the idea that better regulatory measures should be considered for TCS. Currently, TCS is a candidate for substitution given its characterisation as toxic and very bioaccumulative as proposed the BPC. Our results can be useful for the current activities of CoRAP which is evaluating concern on TCS and its use in the European Union.

In conclusion, the PAF method has been successfully used in a number of cost calculations for chemical exposure. In contrast, our results show that the IPRA method requires that uncertainty calculations should be included before its application to other substances in a regulatory context. Our findings clearly demonstrate the pertinence to evaluation of Triclosan costs and probably apply to other substances yet to be scrutinsed.

## Additional files

Additional file 1: Literature search. Details of the literature search method (key words, databases, search method, results). (DOCX 70 kb)

Additional file 2: Calculations of the attributable fraction and IPRA. Excel sheets containing the formulas and the results for the calculations of the attributable fractions, for the chosen endpoints (increased T3 in adolescents, increase in BMI, early pubertal development). (XLSX 2350 kb)

Additional file 3: Proast script for decreased vas deferens. Lines of script used in Proast for calculations of the CED_animal_ for decreased vas deferens. (TXT 4 kb)

Additional file 4: Proast script for decreased T3. Description of data: Lines of script used in Proast for calculations of the CED_animal_ for decreased T3. (TXT 4 kb)

Additional file 5: Proast script for early pubertal development. Lines of script used in Proast for calculations of the CED_animal_ for early pubertal development. (TXT 2 kb)

## Abbreviations

BMD: Benchmark dose modelling
BMI: Body mass index
BMR: Benchmark Response
CED: Critical effect dose
CES: Critical effect size
CMR: Carcinogenic, mutagenic and toxic to reproduction
CoRAP: Community Rolling Action Programme
HR: Hazard ratio
ICED: Individual critical effect doses
IEXP: Probabilistic distribution of individual exposures
IMoE: Individual margins of exposure
IPRA: Integrated probabilistic risk assessment
IQR: Interquartile range
PAF: Population attributable fraction
PBT: Persistent, bioaccumulative and toxic
REACH: Registration, evaluation and authorisation of chemicals
RR: Risk ratio
SD: Standard deviation
SE: Standard error
SEA: Socioeconomic analysis
TCS: triclosan

## Footnotes

1 The European Union (WHO based definition) defines an endocrine-disrupting chemical as an exogenous substance that causes adverse health effects in an intact organism, or its progeny, secondary to changes in endocrine function [3]

2 In the literature, longitudinal studies are usually used in the calculation [4, 39]. But it is possible to use also other type of studies e.g. cross-sectional or case–control studies [4]. It depends on the quality of epidemiological study

3
http://math.tutorvista.com/statistics/z-score-table.html

4 Hazard ratio (HR) is “*an estimate of the ratio of the hazard rate in the treated* versus *the control group.”* ([54], p2787)

5 Assuming normality instead of lognormality for exposure would result in 0 cases

6 @RISK: User’s guide http://www.palisade.com/downloads/documentation/75/EN/RISK7_EN.pdf
