# Supplementary material for: Comparison of methods for calculating the health costs of endocrine disrupters: a case study on triclosan
Source: Environ Health. 2017 Jun 9;16:55. doi: 10.1186/s12940-017-0265-x (PMC5466740; doi:10.1186/s12940-017-0265-x)
Supplement: Supplementary file 1 — Literature search. Details of the literature search method (key words, databases, search method, results). (DOCX 70 kb) [file 12940_2017_265_MOESM1_ESM.docx]

## Additional file 1

## The literature search

We carried a literature review for toxicological, epidemiological and biomonitoring studies. Publications in English were searched in PubMed, Web of science, Google scholar, and Google.

1. **Toxicological studies**:

We used the combination of keywords: “triclosan + in vivo”, “triclosan + toxicity”, “triclosan + toxicological”, “triclosan + endocrine”. We searched in the databases Web of Science and PubMed. The final results (number of the papers) are summarised in Table A.1.

Table A.1. Resulting number of toxicological papers within each key word combination

|  | Triclosan  +  ln vivo  (08.10.2015) | Triclosan  +  Toxicity  (09.10.2015) | Triclosan  +  Toxicological  (12.10.2015) | Triclosan  +  Endocrine  (12.10.2015) |
| --- | --- | --- | --- | --- |
| Web of Science | 233 | 391 | 29 | 320 |
| PubMed | 142 | 280 | 67 | 182 |

Based on the title and on the abstract, we selected the papers that studied the endocrine effects of TCS (Table A.2).

Table A.2. Resulting number of toxicological papers after the first selection

|  | Triclosan  +  ln vivo | Triclosan  +  Toxicity | Triclosan  +  Toxicological | Triclosan  +  Endocrine |
| --- | --- | --- | --- | --- |
| Web of Science | 33 | 50 | 3 | 38 |
| PubMed | 25 | 42 | 19 | 37 |
| Total (WoS+PubMed) | 42 | 53 | 21 | 46 |

We deleted duplicates and obtained a total of 83 papers. Next we applied the following exclusion criteria:

- The effect was not relevant for humans (we have excluded for example studies on the freshwater snail, *Physa acuta* that was done exclusively for measuring environmental impacts of TCS); 33 papers were excluded
- The paper tested a combination of TCS and another substance; 3 papers were excluded
- As the dose-response modelling we used is based on in vivo data, we have excluded in vitro studies; 26 papers were excluded

On the 22 remaining papers we applied QUALICHEM, a quality assessment tool (Maxim, 2014). The scores of this assessment are given in the table below.

Table A.3. List of selected toxicological studies

| **Author** | **Study** | **QUALICHEM score**  **(max = 270)** |
| --- | --- | --- |
| Kummar et al. 2008 | Decreased activity of adenylyl cyclase enzyme and depressed testosterone production | 193 |
| James et al. 2010 | Inhibition of estrogen sulfotransferase activity (the ability of the placenta to supply estrogen to the fetus, and in turn on fetal growth and development) (sheep placenta) | 200 |
| Crofton et al. 2007 | Decreased thyroxine (T4) | 202 |
| Jung et al. 2012 | Estrogenic activity of triclosan in the uterus of immature rats and rat pituitary GH3 cells | 204 |
| Axelstad et al. 2013 | Reduced thyroxine (T4) | 214 |
| Crawford et al. 2012 | Disruption of blastocyst implantation | 221 |
| Paul et al. 2010b | Reduced thyroxine (T4) | 223 |
| Lan et al. 2013 | Decreased daily sperm production (DSP), changed sperm morphology and epi- didymal histopathology | 226 |
| Rodriguez et al. 2010 | Decreased T4, T3 in pregnant rats | 226 |
|  | Lowered sex ratio |  |
|  | Lowered pup bw |  |
|  | Delayed vaginal opening in offspring |  |
| Manservisi et al. 2015 | Altered mammary gland development | 227 |
| Paul et al. 2010a | Decreased T3 and T4 | 230 |
| Paul et al. 2013 | Inhibition of thyroperoxidase enzyme | 231 |
| Paul et al. 2012 | Decreased T4 hormone | 234 |
| Yueh et al. 2014 | Liver fibrogenesis and tumorigenesis | 234 |
| Stoker et al. 2010 | Advanced age of vaginal opening onset | 236 |
|  | Increased uterine weight |  |
|  | Suppressed thyroid hormone |  |
| Kummar et al. 2009 | Perturbed translation of testicular StAR and AR proteins | 240 |
|  | Decreased serum lutenizing hormone (LH), follicle stimulating hormone (FSH), cholesterol, pregnenolone, and testosterone |  |
|  | Reduced sperm production |  |
|  | Reduced testis weight |  |
|  | Reduced seminal vesicle |  |
|  | Reduced ventral prostate |  |
|  | Reduced epididymis |  |
|  | Reduced vas defrentia |  |
| Zorilla et al. 2009 | Decreased total serum thyroxine (T4) | 258 |
|  | Decreased Triiodothyronine (T3) |  |
|  | Increased liver weight |  |
| Foran et al. 2000 | Potentially weakly androgenic (medaka fry Oryzias latipes) | NA (Qualichem is only adapted to in vivo studies) |
| Muth-Kohne et al. 2012 | Morphological effects like edemas, a reduced blood circulation and malformations of head, heart and tail (the zebrafish embryo toxicity test) | NA |
| Fort et al. 2010 | Effect on thyroid hormone - no effect of TCS | NA |
| Fort et al. 2011 | Increased larval growth | NA |
| Pinto et al. 2013 | Significant up-regulation of the sodium–iodide symporter (NIS) and thyroid-stimulating hormone (TSH) | NA |

In this table the studies that were selected for further calculations are marked in green. This selection was done both on the bases on the QUALICHEM score and on the appropriateness of the end-points adressed for the objectives of our project. More precisely we selected those studies that dealt with effects for which we had both toxicological and epidemiological studies available in the literature (which allowed us to compare our two methods for calculating the share of population showing an effect).

1. **Epidemiological studies**

We used the combinations of keywords: “triclosan + longitudinal”, “triclosan + cross-sectional”, “triclosan + case-control”, “triclosan + cohort”, “triclosan + epidemiology”, “triclosan + epidemiologic”, “triclosan + exposure”. We searched in the databases Web of Science and PubMed. The final results (number of the papers) are summarised in table A.4.

Table A.4. Resulting number of epidemiological papers within each key words combination

|  | Triclosan + longitudinal | Triclosan + cross-sectional | Triclosan + case-control | Triclosan + cohort | Triclosan + epidemiology | Triclosan + epidemiologic | Triclosan + exposure |
| --- | --- | --- | --- | --- | --- | --- | --- |
| Web of Science | 30 | 11 | 6 | 20 | 62 | 15 | 546 |
| PubMed | 29 | 12 | 7 | 15 | 72 | 82 | 366 |

We downloaded all papers and deleted duplicates, giving 156 papers (10.9.2015). Based on the abstract and/or the full text of the paper, we selected 15 studies that were clearly related to endocrine effects^[[1]](#footnote-1)^.

The search for biomonitoring studies used the key words triclosan + exposure. This search revealed epidemiological studies that 7 more epidemiological papers were not found with the previous key words.

In total, 15 papers provided epidemiological data dealing with endocrine effects (Table A.5). Among these 15 studies, 5 were used in our calculations. The criteria for including and excluding studies are detailed in Table A.5.

Table A.5. Selection criteria for epidemiological studies (in green, studies included in the calculations)

| **Author** | **Adverse effect** | **Type of study** | **Cohort size** | **Justification for exclusion** |
| --- | --- | --- | --- | --- |
| Allmyr et al. 2009 | Thyroid function - thyroid hormones (TSH, fT3, fT4) | Short-term study (two weeks) | 12 | Small cohort size and insufficient power |
| Buser et al. 2014 | Increased body weight measures and obesity | Cross-sectional | 1298 | The results could not be used in our work (the attributable fraction method), because no association was found between triclosan and the studied body weight outcomes. |
| Buttke et al. 2012 | Age of menarche in adolescent girls | Cross-sectional | 440 | The results could not be used in our work (the attributable fraction method), because triclosan was not significantly (maximal adjusted HR = 1) associated with age of menarche. Also small size of cohort |
| Cullinan et al. 2012 | Thyroid function - thyroid hormones (TSH, fT3, fT4, anti-TGab, TPOab) | Case-control | 132 | Small cohort size. |
| Geens et al.2015 | Thyroid hormones and weight loss in overweight and obese individuals | Cohort study | 194 | Small cohort size. |
| Hond et al. 2015 | Male sub-fertility – inhibin B and LH | Case-control | 163 | Small cohort size. |
| Chen et al. 2013 | Idiopathic male infertility | Case-control | 1590 | The results could not be used in our work (the attributable fraction method), because no significant association was found with idiopathic male infertility (huge range of OR for all semen parameters; e.g. 0.24 – 1.34 OR for semen volume). |
| Koeppe et al. 2013 | Thyroid function – increase of T3 hormone | Cross-sectional | 1831 | Included |
| Lankester et al.2013 | Elavated body mass index | Longitudinal | 4037 | Included |
| Li 2015 | Decrease of body mass index and waist circumference | Cross-sectional | 7964 | Included |
| Philippat et al. 2012 | Offsripng size at birth | Case-control | 288 | The results could not be used in our work (the attributable fraction method), because no association was found with offspring measures at birth; small cohort size |
| Philippat et al. 2014 | Growth in boys | Cohort study | 520 | Small cohort size. |
| Velez et al. 2015 | Decreased fecundity | Cohort study | 1699 | Time to pregnancy based on recall in pregnant women, so not clinically determined. The data is also problematic in that it excludes women who failed to become pregnant. |
| Wolff et al. 2010 | Early pubertal development in girls | Cross-sectional | 1239 | Included |
| Wolff et al. 2015 | Early pubertal development in girls | Cross-sectional | 1239 | Included |

1. **Biomonitoring studies**

We used the key words “triclosan + exposure” and “triclosan + biomonitoring”. We searched in the databases Web of Science and PubMed (22.9.15). The final results (number of the papers) are summarised in the table A.6.

Table A.6. Resulting number of biomonitoring papers within each key words combination

|  | Triclosan exposure | Triclosan biomonitoring |
| --- | --- | --- |
| Web of Science | 546 | 41 |
| PubMed | 366 | 251 |

First, based on the title and on the abstract, we selected those papers that measured triclosan exposure in the human population (blood or urine). In other words we excluded all the papers that measured triclosan in external media (water, different products like toothpaste, soil, etc.). The results after first selection are given in Table A.7.

Table A.7. Resulting number of biomonitoring papers after first selection

|  | Triclosan + exposure | Triclosan + biomonitoring |
| --- | --- | --- |
| Web of Science | 58 | 32 |
| PubMed | 60 | 28 |
| Total (WoS+PubMed) | 68 | 41 |
| Google | 314000 | 19400 |

After deletion of duplicates 73 papers were analysed.

The last selection criterion was exposure measurements restricted to the European populations, which resulted in 16 papers.

This search was combined with a Google search using the same key words. We looked up results displayed on the first 10 Google pages. This search resulted in two additional biomonitoring studies, bringing the total number of studies to 17 (Table A.8).

Table A.8. The list of biomonitoring studies measured TCS within the European population

| **Measured TCS in** | **Measured population (number)** | **Country** | **Author** |
| --- | --- | --- | --- |
| Urine | Children 0-6 years old (21) | Belgium | Pirard et al. 2012 |
|  | Children (30) | Spain | Casas et al. 2011 |
|  | Children 6-11 years old (143) | Denmark | Frederiksen et al. 2013 |
|  | Children 6-11 years old (80) | Sweden | Larsson et al. 2014 |
|  | Children 7-11 years old (21) | Belgium | Pirard et al. 2012 |
|  | Children 8-12 years old (623) | Norway | Bertelsen et al. 2013 |
|  | Adolescents 12-19 years old (22) | Belgium | Pirard et al. 2012 |
|  | Adolescents 12-19 years old (193) | Belgium | Schoeters et al. 2011 |
|  | Pregnant woman (120) | Spain | Casas et al. 2011 |
|  | Pregnant women (191) | France | Philippat et al. 2012 |
|  | Pregnant women (200) | Denmark | Renzy-Martin et al. 2014 |
|  | Pregnant woman | Norway | Bertelsen et al. 2014 |
|  | Women (145) | Denmark | Frederiksen et al. 2013 |
|  | Women (76) | Sweden | Larsson et al. 2014 |
|  | Men (33) | Denmark | Lassen et al. 2013 |
|  | Adults 20-39 years old (22) | Belgium | Pirard et al. 2012 |
|  | Adults 40-59 years old (23) |  |  |
|  | Adults more then 60 years old (22) |  |  |
|  | Population 6 - 64 years old | Germany | Moos et al. 2014 |
|  | Population 2.5 - 85 years old (100) | Greece | Asimakopoulos et al. 2014 |
|  | Population 18-84 years old (383) | Belgium | Geens et al. 2015 |
| Human milk | Women (5) | Sweden | Adolfsson et al. 2002 |
|  | Mothers (9) | Sweden | Allmyr et al. 2006 |
|  |  |  |  |
| Plasma | Mothers (9) | Sweden | Allmyr et al. 2006 |
|  |  |  |  |
|  | Adults (12) exposed for 14 days to toothaste with TCS | Sweden | Allmyr et al. 2009 |
|  |  |  |  |
|  |  |  |  |

# References

Adolfsson-Erici M, Pettersson M, Parkkonen J, et al. (2002) Triclosan, a commonly used bactericide found in human milk and in the aquatic environment in Sweden. *Chemosphere* 46: 1485-1489.

Allmyr M, Adolfsson-Erici M, McLachlan MS, et al. (2006) Triclosan in plasma and milk from Swedish nursing mothers and their exposure via personal care products. *Science of the Total Environment* 372: 87-93.

Allmyr M, Panagiotidis G, Sparve E, et al. (2009) Human exposure to triclosan via toothpaste does not change CYP3A4 activity or plasma concentrations of thyroid hormones. Basic & Clinical Pharmacology & Toxicology 105: 339-344.Asimakopoulos AG, Thomaidis NS, Kannan K (2014) Widespread occurrence of bisphenol A diglycidyl ethers, p-hydroxybenzoic acid esters (parabens), benzophenone type-UV filtres, triclosan, and triclocarban in human urine from Athens, Greece. *Science of the Total Environment* 470-471:1243-1249.

Axelstad M, Boberg J, Vinggard AM, et al. (2013) Triclosan exposure reduces thyroxine levels in pregnant and lactating rat dams and in directly exposed offspring. *Food and Chemical Toxicology* 59: 534-540.

Bertelsen RJ, Engel SM, Jursko TA; et al. (2014) Reliability of triclosan measures in repeated urine samples from Norwegian pregnant women. *Journal of Exposure Science and Environmental Epidemiology* 24(5): 517-521.

Bertelsen RJ, Longnecker MP, Løvik M, et al. (2013) Triclosan exposure and allergic sensitization in Norwegian children. *Allergy* 68: 84-91.

Buser MC, Murray HE, Scinicariello F (2014) Association of urinary phenols with increased body weight measures and obesity in children and adolescents. *The Journal of Pediatrics* 165: 744-749.

Buttke DE, Sircar K, Martin C (2012) Exposures to endocrine-disrupting chemicals and age of menarche in adolescent girls in NHANES (2003-2008). *Environmental Health Perspectives* 120: 1613-1618.

Casas L, Fernandez MF, Llop S, et al. (2011) Urinary concentrations of phthalates and phenols in a population of Spanish pregnant women and children. *Environment International* 37: 858-866.

Chen M, Tang R, Fu G, et al. (2013) Association of exposure to phenols and idiopathic male infertility. *Journal of Hazardous Materials* 250(251): 115-121.

Crawford BR, Catanzaro Dd (2012) Disruption of blastocyst implantation by triclosan in mice: Impacts of repeated and acute doses and combination with bisphenol-A. *Reproductive Toxicology* 34: 607-613.

Crofton KM, Paul KB, et al. (2007) Short-term in vivo exposure to the water contaminant triclosan: Evidence for disruption of thyroxine. *Environmental Toxicology and Pharmacology* 24: 194-197.

Cullinan MP, Palmer JE, Carle AD, et al. (2012) Long term use of triclosan toothpaste and thyroid function. *Science of the Total Environment* 416: 75-79.

Foran CM, Benett ER, Benson WH (2000) Developmental evaluation of a potential non-steroidal estrogen: triclosan. *Marine Environmental Research* 50:153-156.

Fort DJ, Mathis MB, Hanson W, et al. (2011) Triclosan and thyroid-mediated metamorphosis in anurans: Differentiating growth effects from thyroid-driven metamorphosis in Xenopus laevis. *Toxicological Science* 121(2): 292-302.

Fort DJ, Rogers RL, Gorsuch JW, et al. (2010) Triclosan and anuran metamorphosis: No effect on the thyroid-mediated metamorphosis in Xenopus laevis. *Toxicological Sciences* 113(2): 392-400

Frederiksen H, Nielsen JKS, Morck TA, et al. (2013) Urinary excretion of phthalate metabolites, phenols and parabens in rural and urban Danish mother-child pairs. *International Journal of Hygiene and Environmental Health* 216: 772-783.

Geens T, Dirtu AC, Dirinck E, et al. (2015) Daily intake of bisphenol A and triclosan and their association with anthropometric data, thyroid hormones and weight loss in overweight and obese individuals. *Environment International* 76: 98-105.

Hond ED, Tournaye H, Sutter PD, Ombelet W et al. (2015) Human exposure to endocrine disrupting chemicals and fertility: A case-control study in female subfertility patients. *Environment International* 84:154-160.

James MO, Li W, Summerlot DP, et al. (2010) Triclosan is a potent inhibitor of estradiol and estrone sulfonation in sheep placenta. *Environment International* 36: 942-949.

Jung EM, An BS, Choi KC, et al. (2012) Potential estrogenic activity of triclosan in the uterus of immature rats and rat pituitary GH3 cells. *Toxicology Letters* 208: 142-148.

Koeppe ES, Ferguson KK, Colacino JA, et al. (2013) Relationship between urinary triclosan and paraben concentrations and serum thyroid measures in NHANES 2007-2008. *Science of the Total Environment* 445(446): 299-305.

Kumar V, Balomajumder C, Roy P (2008) Disruption of LH-induced testosterone biosynthesis in testicular Leydig cells by triclosan: Probable mechanism of action. *Toxicology* 250: 124-131.

Kumar V, Chakraborty A, Kural MR, et al. (2009) Alternation of testicular steroidogenesis and histopathology of reproductive system in male rats treated with triclosan. *Reproductive Toxicology* 27: 177-185.

Lan Z, Kim TH, Bi KS, et al. (2013) Triclosan exhibits a tendency to accumulate in the epididymis and shows sperm toxicity in male Sprague-dawley rats. *Environmental Toxicology* 83-91.

Lankester J, Patel C, Cullen MR, et al. (2013) Urinary triclosan associated with elevated body mass index in NHANES. PLoS ONE 8(11):e80057. doi:10.1371/journal.pone.0080057

Larsson K, Björklund KL, Palm B, et al. (2014) Exposure determinants of phthalates, parabens, bisphenol A and triclosan in Swedish mothers and their children. *Environment International* 73: 323-333.

Lassen TH, Frederiksen H, Jensen TK, et al. (2013) Temporal variability in urinary excretion of bisphenol A and seven other phenols in spot, morning, and 24-h urine samples. *Environmental Research* 126: 164-170.

Li S, Zhao J, Wang G, et al. (2015) Urinary triclosan concentrations are inversely associated with body mass index and waist circumference in the US general population: Experience in NHANES 2003-2010. *International Journal of Hygiene and Environmental Health* 218: 401-406.

Manservisi F, Gopalakrishnan K, Tibaldi E, et al. (2015) Effect of maternal exposure to endocrine disrupting chemicals on reproduction and mammary gland development in female Sprague-Dawley rats. *Reproductive Toxicology* 54: 110-119.

Maxim L, Van der Sluijs J (2014) Qualichem in vivo: A tool for assessing the quality of in vivo studies and its application for Bisphenol A. PLOS One. [doi : 10.1371/journal.pone.0087738](http://dx.doi.org/10.1371/journal.pone.0087738)

Moos RK, Angerer J, Wittsiepe J, et al. (2014) Rapid determination of nine parabens and seven other environmental phenols in urine samples of German children and adults. *International Journal of Hygiene and Environmental Health* 217: 845-853.

Muth-Kohne E, Wichmann A, Delov V, et al. (2012) The classification of motor neuron defects in the zebrafish embryo toxicity test (ZFET) as an animal alternative approach to assess developmental neurotoxicity. *Neurotoxicology and Teratology* 34: 413-424.

Paul KB, et al. (2010a) Short-term exposure to triclosan decreases thyroxine in vivo via upregulation of hepatic catabolism in young long-evans rats. *Toxicological Sciences* 113(2): 367-379.

Paul KB, et al. (2010b) Developmental triclosan exposure decreases maternal and neonatal thyroxine in rats. *Environmental Toxicology and Chemistry* 29(12): 2840-2844.

Paul KB, et al. (2012) Developmental triclosan exposure decreases maternal, fetal, and early neonatal thyroxine: A dynamic and kinetic evaluation of a putative mode-of-action. *Toxicology* 300: 31-45.

Paul KB, et al. (2013) Evidence for triclosan-induced activation of human and rodent xenobiotic nuclear receptors. *Toxicology in Vitro* 27: 2049-2060.

Philippat C, Mortamais M, Chevrier C, et al. (2012) Exposure to phthalates and phenols during pregnancy and offspring size at birth. *Environmental Health Perspectives* 120(3): 464-470.

Philippat C, Botton J, Calafat AM, et al. (2014) Prenatal exposure to phenols and growth in boys. *EPIDEMIOLOGY* 25(5): 625-635.

Pinto P, Guerreiro EM, Power DM (2013) Triclosan interferes with the thyroid axis in the zebrafish (Danio rerio). *Toxicology Research* 2: 60-69.

Pirard C, Sagot C, Deville M, et al. (2012) Urinary levels of bisphenol A, triclosan and 4-nonylphenol in a general Belgian population. *Environment International* 48: 78-83.

Renzy-Martin KT, et al. (2014) Current exposure of 200 pregnan Danish women to phthalates, parabens and phenols. *Reproduction* 147: 443-453.

Rodriguez PEA, Sanchez MS (2010) Maternal exposure to triclosan impairs thyroid homeostasis and female pubertal development in wistar rat offspring. *Journal of Toxicology and Environmental health, Part A*. 73: 1678-1688.

Schoeters G, Colles A, Den Hond E, Croes K, Vrijens J, Baeyens W, et al. The Flemish Environment and Health Study (FLEHS) — second survey (2007–2011): establishing reference values for biomarkers of exposure in the Flemish population. In: Knudsen LE, Merlo DF, editors. Biomarkers and human biomonitoring volume 1: ongoing programs and exposuresR Soc Med; 2011. p. 135–65.

Stoker TA, Gibson EK, Zorilla LM (2010) Triclosan exposure modulates estrogen-dependent responses in the female wistar rat. *Toxicological Sciences* 117(1): 45-53.

Velez MP, Arbuckle TE, Fraser WD (2015) Female exposure to phenols and phthalates and time to pregnancy: the maternal-infant research on environmental chemicals (MIREC) study. *Environment and Epidemiology* 103(4): 1011-1020.

Wolff MS, et al. (2010) Investigation of relationships between urinary biomarkers of phytoestrogens, phthalates, and phenols and pubertal stages in girls. *Environmental Health Perspectives* 118(7): 1039-1046.

Wolff MS, et al. (2015) Environmental phenols and pubertal development in girls. *Environment International* 84: 174-180.

Yueh MF, et al. (2015) The commonly used antimicrobial additive triclosan is a liver tumor promoter. *Proceedings of the National Academy of Sciences* 112(2): 17200-17205.

Zorilla LM, et al. (2009) The effects of triclosan on puberty and thyroid hormones in male wistar rats. *Toxicological Sciences* 107(1): 56-64.

1. We have defined endocrine effect as being the effects that is caused by a substance, “*either natural or synthetic, which through environmental or inappropriate developmental exposures alters the hormonal and homeostatic systems that enable the organism to communicate with and respond to its environment.” (Endocrine Society)* [↑](#footnote-ref-1)
